# Learning-Based Methods of Perception and Navigation for Ground Vehicles in Unstructured Environments: A Review

**DOI:** 10.3390/s21010073

**Published:** 2020-12-25

**Authors:** Dario Calogero Guastella, Giovanni Muscato

**Affiliations:** Dipartimento di Ingegneria Elettrica, Elettronica e Informatica, Università degli Studi di Catania, Viale A. Doria 6, 95125 Catania, Italy; gmuscato@dieei.unict.it

**Keywords:** unmanned ground vehicle navigation, end-to-end navigation, terrain traversability analysis, machine learning paradigms, deep learning for robotics, off-road navigation

## Abstract

The problem of autonomous navigation of a ground vehicle in unstructured environments is both challenging and crucial for the deployment of this type of vehicle in real-world applications. Several well-established communities in robotics research deal with these scenarios such as search and rescue robotics, planetary exploration, and agricultural robotics. Perception plays a crucial role in this context, since it provides the necessary information to make the vehicle aware of its own status and its surrounding environment. We present a review on the recent contributions in the robotics literature adopting learning-based methods to solve the problem of environment perception and interpretation with the final aim of the autonomous context-aware navigation of ground vehicles in unstructured environments. To the best of our knowledge, this is the first work providing such a review in this context.

## 1. Introduction

Unmanned Ground Vehicles (UGVs) are probably the first type of modern autonomous mobile platforms to have appeared in robotics research. A first example of an autonomous intelligent mobile wheeled robot can even be dated back to 1949, when W. Grey Walter designed and built the so-called electro-mechanical tortoises, driven by biologically inspired control [1]. Since then, field robotics research has reached several milestones ranging from first semi-autonomous planetary rovers [2] to autonomous off-road driverless cars [3]. However, in spite of the rather long history of ground mobile robots, there are still several open issues in the field of autonomous navigation of such vehicles in so-called *unstructured environments*. According to [4], unstructured environments are those environments which have not been previously modified in order to simplify the execution of a task by a robot. In this work we refer to those operating environments lacking clearly viable paths or helpful landmarks in supporting the autonomous navigation of the vehicle.

A significant body of literature on autonomous ground navigation has been mainly focused on indoor structured environments. In recent years, an important stimulus on outdoor environments has come from the research on self-driving cars, which has now become a well-established reality [5,6]. As opposed to the indoor scenario, where autonomous navigation has already reached a high technology readiness level (TRL), the goal of having a reliable fully-autonomous car cannot yet be considered accomplished, even in the urban scenario where lane markings or curbs can support the identification of drivable paths. Clearly, other aspects have to be addressed in this context, mainly arising from traffic rules and, more importantly, the coexistence in the same operating environment of many other external agents and vehicles (of different types).

In this survey we want to focus on the problem of autonomous ground vehicle navigation in unstructured environments, which is both challenging and crucial for the deployment of this type of vehicle in real-world applications. Several well-established communities in robotics research deal with these types of scenarios such as search and rescue robotics, planetary exploration, and agricultural robotics to name a few. It can be easily figured out how *perception* plays a crucial role in this context, since it provides the necessary information to make the vehicle aware of its own status and its surrounding environment to properly interact with. The term perception encloses the overall sensing system, including both the sensing devices and the solutions for high-level information extraction from the raw data.

According to this definition, we will present a review on the recent contributions in the robotics literature adopting *learning-based* methods to solve the problem of environment perception and interpretation with the final aim of autonomous *context-aware* navigation of ground vehicles in unstructured environments. In this manner we want to focus on those navigation solutions not relying on the detection of artificial landmarks or typical features of structured environments, such as corridors’ boundaries in mazes or indoor office-like environments, or road lanes, and so on. Several examples are available in the literature for these scenarios [7,8,9,10,11,12].

Furthermore, although machine learning has been adopted for decades in off-road autonomous navigation, these methods have recently gained new momentum thanks to the generally improved computing power and the introduction of novel types of sensors available on board the vehicles. Moreover, the recent breakthrough of artificial intelligence and deep learning methods has pushed advancements on several learning paradigms, thus opening up novel solutions to interpret sensory data and, more importantly in the robotics context, to define control policies. These methods represent a relevant research stream which must be further explored.

The main contribution of this work is to provide a broad high-level review on the main approaches to the autonomous navigation of ground vehicles in the specific *learning* realm and in the *unstructured environment* context. To the best of our knowledge, this is the first work attempting such a review aiming at organising the wide spectrum of approaches mentioned above. Clearly, we do not claim to provide a comprehensive survey of all the solutions reported in the literature, as this would require an almost unbearable effort. By means of this review, we wish to help researchers working on this topic to be able to obtain a clear overview of the most relevant methods currently available in the literature, as well as to easily identify the most useful approach for the problem at hand.

The remainder of this paper is structured as follows: Section 2 will provide a brief overview of other related surveys and the boundaries of the present work. Section 3 will introduce the criteria adopted to classify the works reported in the following sections, while Section 4 and Section 5 will go into the details of each category. A wide Discussion is reported in Section 6, including open questions for research, in order to provide stimuli and insights for future developments and investigations. Finally, Section 7 concludes the paper.

## 2. Related Works and Survey Boundaries

Several surveys and reviews can be found in the literature related to the navigation of ground robots, both in structured and unstructured environments, or related to general machine learning methods for robotics. The works by Chhaniyara et al. and Papadakis [13,14] provide an early overview on so-called *terrain traversability analysis* (TTA) methods for UGV navigation over rough terrains. Terrain traversability analysis can be roughly referred to as the problem of estimating the difficulty of driving through a terrain for a ground vehicle. This approach is clearly conceptually different from a classical obstacle avoidance problem, since most of the time no physical obstacle is actually present, whereas a trade-off between different paths has to be performed. Chhaniyara et al. focus their review on rovers for planetary exploration and soil mechanical property characterization. On the other hand, Papadakis provides a general overview on TTA ranging from classical geometry-based approaches, passing through proprioceptive-based methods and appearance-based methods and concluding with hybrid approaches. Although early learning-based methods are presented, the paper was written before the recent breakthrough of deep learning, especially in computer vision, which has also brought the sudden proliferation of alternative methods to classical machine learning approaches.

The work by Grigorescu et al. [15] deals specifically with deep learning methods instead. A detailed perspective on the context of autonomous cars is given, including decision making solutions, neural networks architectures and employed hardware. However, it is essentially placed in the urban on-road scenario. Other very recent surveys about deep learning methods and applications are available in the literature still in the self-driving cars domain [16,17]. However, the work by Kuutti et al. [16] is mainly concerned with vehicle control rather than perception, whereas the one by Ni et al. [17], similarly to [15], presents a wide review on the deep learning-based solutions used in self-driving urban vehicles tasks, such as object detection, lane recognition, and path planning. Another work still focused on deep learning-based control techniques, both for manipulation and navigation, is the one by Tai et al. [18].

A remarkable review on general learning methods for mobile robots navigation is given by Wulfmeier [19]. This work reports a broad discussion on the adoption of machine learning for each module of the navigation pipeline, from perception and mapping to planning and control. However, also this work is mainly oriented towards the urban scenario.

A very recent work by Hu et al. [20] provides an interesting overview on sensing technologies and sensor fusion techniques for off-road environments. Although perception is the core topic of this work, it does not put emphasis on the recent and novel learning based methods. Finally, other reviews deal with specific learning paradigms, such as learning from demonstration [21,22] or reinforcement learning [23], without a particular attention to navigation.

**Boundaries**

The main goal of this paper is to summarize and classify those solutions leveraging machine learning for the perception and comprehension of unstructured environments for the autonomous navigation of unmanned ground vehicles. According to this goal and for the sake of conciseness, this review purposefully does not include:

- multi-robot approaches, as in those works many other aspects arise, ranging from the shared knowledge of the surrounding world to the multi-platform coordination and management;
- works exclusively based on environment interaction or proprioceptive data (e.g., analysis of vehicle vibrations or attitude). We focus on *environment perception* in order to define a traversable path *before* actually crossing it;
- works purely dealing with path planning algorithms. We want to include only those solutions where the environments characteristics are taken into account;
- platform-specific works not including terrain information, such as prediction of the vehicle’s power consumption or low-level traction or motion control.

Recalling the well-known *sense-plan-act* paradigm in autonomous robot navigation, we essentially include all those works where at least the very first step, i.e., *sense*, is included. Therefore, we include those works performing planning, without moving further to the motion action, and even the solutions embedding all three aspects in a monolithic solution. There is clearly an unavoidable and partial overlap between perception and control, although this distinction is becoming more and more often evanescent.

Although this approach might look excessively broad and some approaches may even appear distant, we believe that since the reported solutions share a common starting point, i.e., the unstructured nature of the perceived environment, it is possible to identify some aspects which can be actually adapted to the different consequent tasks, namely planning, and low-level control. We argue that the side-by-side comparison of such solutions can provide a set of useful insights for the reader.

Regarding the survey methodology, the works included in this review have been selected among the papers collected over the last few years from the main scientific databases (namely Scopus, Web of Science and Google Scholar). The related search terms are *ground vehicle*, *unstructured environments*, *learning*, and *traversability*. Moreover, a further thorough research has been performed from the references included in the reported works.

## 3. Categorization

Within the boundaries described in the previous section it is possible to identify two main broad categories of learning-based methods for autonomous navigation in unstructured environments:

1. methods based on *terrain traversability analysis* (TTA)
2. *end-to-end* methods

Evidently, these two categories are deeply different. As already partially introduced in Section 1 by traversability we refer to the capability of a ground vehicle to stably reach a terrain region; this capability being dependent on (1) the terrain model, (2) the vehicle model, (3) the kinematic constraints of the vehicle and, in some cases, (4) some optimization criteria [14]. Methods relying on TTA can be in turn divided into two main approaches: (1) *regression of traversal costs* and (2) *terrain classification*. Similarly to machine learning lexicon, the former provides a continuous measure encoding the traversal cost (i.e., the expected difficulty the UGV would experience during the traversal); whereas the latter aims at identifying the terrain classes with navigation-relevant properties in the environment. Besides this rigid distinction, there are also some works based on a mixed regression-classification approach for TTA. In the context of an autonomous navigation framework, a further processing step is needed for handling the outcome of the traversability assessment, in order to translate such an assessment into actual motion.

**Figure 1:**
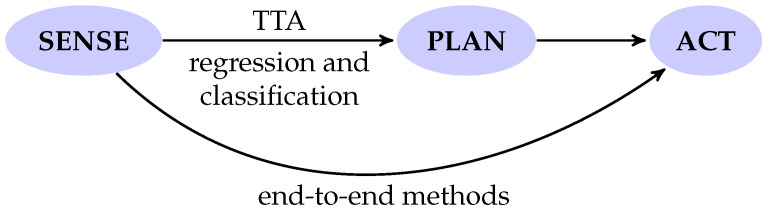
Terrain traversability analysis (TTA) and end-to-end methods comparison.

**Figure 2:**
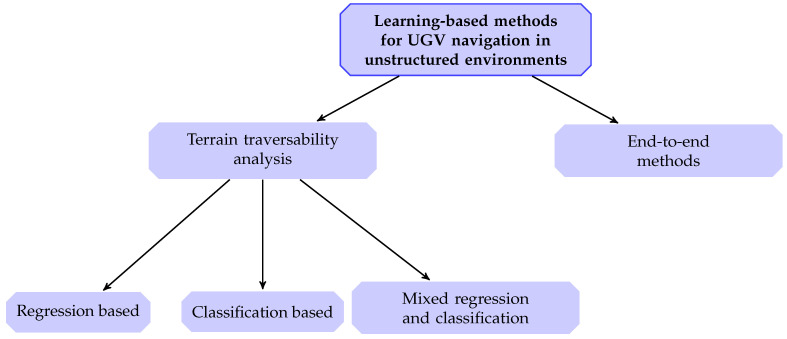
Categorization method adopted in this survey. (UGV—Unmanned Ground Vehicle).

Conversely, end-to-end methods directly map raw environment perception and/or vehicle state (i.e., raw exteroceptive and proprioceptive sensory data) to navigation control actions, thus fusing perception and control of the navigation framework into a single block. From this perspective, end-to-end methods may also be seen as a particular case of TTA-based methods, as they include both TTA and control. Although end-to-end methods might appear to offer a simpler and immediate solution, they also have some drawbacks as will be discussed in Section 6. Recalling again the sequential sense-plan-act pipeline, Figure 1 depicts where TTA and end-to-end methods are placed. Finally, for both of them we distinguish between classical learning and deep learning-based solutions. In Figure 2 a scheme representing the adopted categorization method is reported. In the following sections we will make extensive use of acronyms, especially in summary tables. To help the reader, we reported a list of them in the Abbreviations section, at the end of this manuscript.

## 4. Terrain Traversability Analysis

### 4.1. Regression of Traversal Costs

As already introduced, terrain traversability analysis based on regression aims at estimating the *traversal cost* of a terrain patch, somehow encoding the difficulty that the vehicle would encounter while crossing the considered terrain patch. Most of the time the goal is to identify a map of costs (or cost map) of the surrounding environment, on top of which path planning or motion control can be performed.

#### 4.1.1. Solutions Based on Classical Machine Learning

Several early approaches to regression-based TTA lie in the *learning from demonstration* (LfD) context. These methods allow us to retrieve from the expert’s demonstrations a function mapping a set of hand-crafted features, extracted from the sensory data, to a traversal cost, which is a nontrivial problem. From such a function it is then possible to derive a cost map for motion planning. Even though most of these LfD-based works do not explicitly refer to traversability analysis, these methods, enhanced through deep learning, have been recently proposed to address specifically traversability analysis for path planning in large urban and semi-structured scenarios [19,24].

An example of early work based on LfD is given by Zucker et al. [25], who propose a solution for a quadrupedal robot based on ranking support vector machines (SVMs) [26,27]. In this work a vector of terrain geometry features is determined by a local quadratic regression of terrain patches around footholds suggested by an expert. This vector is used to derive a utility function encoding the terrain cost function. The height map of a testbed rough terrain is a priori known and the position of the vehicle inside the testing scenario is acquired through a motion capture system.

With the same testbed in a previous work, Ratliff et al. [28] propose the boosted maximum margin planning (MMP) algorithm as learning from demonstration paradigm. Also in this case the goal is to derive a heuristic to be adopted by the footstep planner of the quadruped. From the expert sampled demonstration trajectory, a set of morphological terrain features is extracted.

Still in [28] the algorithm is successfully used also for training a local obstacle avoidance planner, based on sensory data from on-board devices, i.e., laser scanners, RGB and Near Infrared (NIR) cameras providing geometry and appearance information respectively. From these sensors a set of features for each cell of a local grid map surrounding the vehicle are derived, including the ground plane elevation and slope, the detected laser points density and the average color.

The same experimental setup is adopted by Kolter et al. [29,30], still in the framework of the DARPA Learning Locomotion program [31]. It still consists of a learning from demonstration approach, which can be seen as a kind of hierarchical inverse reinforcement learning (IRL). Also in this case a set of local features (virtually encoding roughness and slope) are derived from the patch of terrain height map around the quadruped’s footstep. The footsteps and trajectories suggested by the expert are used to learn the linear combination of the terrain features, i.e., the footstep cost, thus establishing a trade-off among them. Then a cost map is derived and used by the low-level footstep planner, whereas the aggregation of each foot cost is used by the high-level body path planner.

Silver et al. [32] propose a solution based on MMP with non-linear cost functions [33], combining several features from on-board sensors, i.e., 2D light detection and rangings (LIDARs) and a stereo camera. Among these features are the obstacle heights and LIDAR point density. The learned cost map, derived from expert demonstration and based on the detected perceptual features, is used by the two-level planner of a six-wheel skid steering vehicle.

Beyond LfD methods, an unsupervised method is adopted by Faigl and Prágr [34] for traversability cost maps regression. In particular self-organizing maps are proposed, to predict three traversal costs, i.e., instantaneous power consumption, maximum achievable forward velocity, and attitude stability, based on the morphology and the appearance of the terrain, acquired through an RGB-D camera. Furthermore, during the learning phase the predicting model is also fed with the traversal costs actually experienced by the robot during the traversal. Once learned the model is deployed for predicting the cost from the exteroceptive data only.

In a similar fashion, ekhti and Kobayashi [35] train a Gaussian process regressor (GPR) in order to predict vehicle vibration (as a measure of terrain traversability) while moving over the terrain, combined with terrain textures features detected by processing images from an on-board RGB camera. Also in this case, the regressor is trained with proprioceptive data (i.e., accelerometer data) acquired during the traversal, whereas on-line traversal cost regression is based on the incoming RGB image only.

An off-road navigation strategy is presented by Quann et al. [36] based on probabilistic energy cost prediction. A Gaussian process regressor realizes a mapping from current robot pose, terrain slope (along robot motion direction) and grayscale satellite imagery to power consumption. The map of energy costs is eventually used for predicting the cumulative power consumption during a path traversal.

Table 1 summarizes the works introduced in this section. For the sake of clarity, *input data* refers to the data actually needed by the regression solution at deployment time, i.e., when the vehicle is traversing the terrain, as opposed to the data provided during training.

#### 4.1.2. Solutions Based on Deep Learning

Also in the context of deep learning-based traversal cost regression most of the works belong to the learning from demonstration paradigm, especially to the recent wave of Deep Inverse Reinforcement Learning (DIRL) and maximum entropy DIRL (ME-DIRL) [37,38]. In this case, the traversability cost map is indirectly obtained as the negative reward function learned from the expert demonstrations.

The work by Zhang et al. [39], even though it is not specifically conceived for terrain traversability analysis, presents a two-stage network architecture to predict from a third-person perspective autonomous cars’ path while driving off-road. The approach is framed in a maximum entropy DIRL solution to include both the environmental context and the kinematic of the vehicle in the reward function modeled by the two-stage network. In particular, the first stage is a four-layer fully convolutional network (FCN), which takes as inputs a set of statistical indexes of the colored point cloud acquired during the expert demonstrations and outputs several feature maps, somehow encoding traversability measures of the environment (at different levels). These maps are then combined with the second stage related to the kinematic aspect of the vehicle to perform the original task. Hence, a global cost map from such features is not established, which would represent the final traversability cost map.

A ME-DIRL approach is proposed also by Zhu et al. [40]. Also in this case vehicle kinematics is taken into account (through two novel convolutional network architectures), whereas as sensory input 3D laser scans are used to create 2.5D local grid maps of the surrounding environment. Expert trajectories are recorded by GPS and provided along with the grid map. The reward structure is modeled by a 5-layer FCN. The authors successfully trained four different types of driving behaviors, capable of dealing with negative obstacles as well.

Pflueger et al. [41] propose an interesting alternative method to ME-DIRL, based on inverse reinforcement learning combined with a soft value iteration network (SVIN), as defined by the authors. The network is trained feeding as input a stack of images (including the satellite imagery of the considered terrain), a map highlighting the goal position and other optional inputs, such as elevation data, hyper-spectral imagery, and terrain roughness. Expert demonstrations are shown as paths over the provided images. During deployment, thanks to the policy implied by the value function, a global path planning can be performed. Moreover, the value map delivered by the value iteration module can be combined with a lower level planner.

In Table 2 a summary of the works introduced in this section is reported, along with the information on the adopted deep neural network architectures.

### 4.2. Classification of Terrain Type

Another widely explored approach in addressing navigation in unstructured environments through traversability analysis is based on assigning terrain areas to specific classes.

#### 4.2.1. Solutions Based on Classical Machine Learning

Santamaria-Navarro et al. [42] propose a classification method of 3D point clouds acquired by a laser scanner. A binary classification is performed via Gaussian process classification as well as regression (with a final value thresholding). The classification is trained by providing positive examples only (i.e., traversable areas), taking into account the robot footprint and 3D point clouds acquired while the vehicle is driven by a human operator. In particular, the point cloud is projected in a grid map and for each cell terrain slope and roughness are derived through principal component analysis (PCA)

Suger et al. [43] present a similar solution for mobile robots equipped with a 3D LIDAR. A 2D grid map of feature vectors is considered, derived from the perceived 3D point clouds, including both geometrical features derived from PCA and the laser reflection values. The training data are acquired while a user manually operates the ground vehicle. In view of the scarce positive examples acquired with this strategy, two approaches to train a classifier are adopted [44,45]. Both of them produced successful results with two different types of vehicles and different real-world scenarios.

In their work, Lee et al. [46] perform a three-step traversability classification. First, an automatic region labeling is carried out by segmenting the image into visually coherent areas (superpixels). The unknown regions in front of the vehicle are labeled as drivable while the vehicle is in motion. Then training samples are extracted (i.e., color and texture histograms) and are provided to the second stage, an incremental model learning stage. It is a self-supervised learning architecture referred to as incremental non-parametric Bayesian clustering. The final stage consists of a binary region classification based on a k-nearest neighborhood (kNN) algorithm.

Ahtiainen et al. [47] propose an interesting approach to deal with vegetated environments, where most geometry-based methods misclassify dense vegetation as non-traversable by using a 3D LIDAR. A probabilistic voxel grid map, that the authors refer to as a normal distributions transform traversability map, is used, including geometrical information, the permeability, and the reflectivity for each cell. Two classifiers based on non-linear support vector machines are trained to perform a binary classification.

In the work by Sock et al. [48] two probabilistic maps are independently built from RGB camera images and 3D LIDAR scans and combined through Bayesian fusion. A linear SVM classifier is chosen to binary classify RGB images depending on a set of visual features. Training data are collected from the first image assuming that the terrain in front of the vehicle footprint is flat and traversable (i.e., it is a positive example), whereas those terrain blocks with the furthest mean RGB intensity from the mean intensity of the positive samples are marked as negative samples. After that, traversable region detection is performed on the following frames. The pseudo-probability value is obtained by giving classification results to a sigmoid function. Laser scans from the 3D LIDAR are used to build a 2.5D elevation model instead. From this model a map of slopes is derived and, then, a map of traversability scores, through an empirical exponential function. Finally the two maps are spatially and temporally aligned to be fused and to obtain the cumulative traversability map, treated as a Markov random field.

Hewitt et al. [49] present a terrain classifier for a micro-rover for planetary exploration. The classifier takes as inputs statistics (features) of 2D scans from the on-board LIDAR, by dividing the environment into cells. The main statistics derived for each cell are the height mean value and variance, and the number of LIDAR points. Rover pose is taken into account as well. The binary classification on cloud points is performed by a multilayer perceptron (MLP) trained through an extended Kalman filter. A relevant part of this work is the wide adoption of simulated data for training, to facilitate the work of data labeling.

A computer vision pipeline for traversable path detection from on-board images is proposed by Deng et al. in [50]. The pipeline consist of estimating texture orientations, in order to estimate the vanishing point through line segment candidates voting, and finally constructing a multivariate Gaussian appearance model by selecting a sample region inside the traversable path. This model is used for classifying the image into traversable and non-traversable areas through segmentation.

Bellone et al. [51] propose a binary classification through a support vector machine. Also in this case a combination of geometric and visual features are derived from the 3D point cloud obtained from an on-board stereo camera. The authors introduce a particular geometric normal-based feature, by means of which they are able to enhance reliability and robustness of the classification. The work also reports several trials with four different kernel functions for the SVM.

Kingry et al. [52] propose a multilayer perceptron capable of classifying six terrain types, starting from color and textural features derived from a single RGB camera image. The paper discusses several approaches with a variety of visual feature sets.

Several terrain classification methods have been tested by Martínez et al. [53], ranging from decision trees to random forests. The authors trained all the classifiers on synthetic 3D laser scanner data generated in simulation and tested them on data acquired from the on-board 3D LIDAR of a skid steering vehicle moving in the real environment. The classifier that performed better across the two domains was the random forest (RF). The classification is performed on a set of spatial features derived for each point of the acquired point cloud, namely the minimum height coordinate among all the neighbours, and through PCA, the normal orientation and roughness.

Schilling et al. [54] propose a terrain classification approach with three terrain traversability classes: safe, risky, and obstacle. The classification process relies on two complementary pipelines for extracting geometric and semantic features. Environment geometric features (slope, max height, and roughness) are derived by processing 3D LIDAR point cloud again through PCA. In parallel, a fully convolutional network is trained to perform semantic segmentation from the camera image. Both these sets of features are then fused, through a suitable concatenation approach, in order to obtain the final terrain classification by using a random forest classifier. It is worth noting that although this work uses an FCN for semantic segmentation, the very final classification is performed by a random forest. This justifies the location of this work in this section.

In Table 3, the works discussed in this section are summarized.

#### 4.2.2. Solutions Based on Deep Learning

In the context of classification-based terrain traversability analysis a considerable boost has been provided in recent years by advancements in deep learning, especially in computer vision and image segmentation.

One of the first works in this direction is the Soil Property and Object Classification (SPOC) tool for planetary rovers presented by Rothrock et al. [55]. It consists of a fully convolutional network, allowing the image segmentation of terrain types (e.g., sand, bedrock, etc.) from either orbital or ground-acquired images. The proposed solution has been adopted both to identify a feasible landing site for the Mars 2020 rover mission and to build a slip prediction model, by correlating predicted terrain classes with observed wheel slip and slope angles by the Curiosity rover.

An interesting terrain segmentation strategy based on multispectral imagery is proposed by Valada et al. [56], in order to enhance segmentation robustness in real-world light-varying outdoor environments. The paper deals with the issue of how to fuse the various channels (namely RGB, depth and NIR images) proposing early and late fusion architectures in the fully convolutional network used.

Chavez-Garcia et al. [57] address traversability analysis as a height map binary classification through a convolutional neural network (CNN). The height map is related to the terrain patch in front of the vehicle. Training data are created through procedural generation in simulation, over synthetic terrains (with specific features). A wheeled vehicle running at a constant velocity through the environment is simulated and height maps of the terrain patches surrounding the vehicle are extracted. A distance measure between consecutive robot poses acquired from the simulation is used to label each terrain patch as traversable or not.

A semantic 3D mapping approach is reported by Maturana et al. [58] to support autonomous off-road navigation of a UGV. The real-time mapping strategy provides a 2.5D grid map centered on the vehicle frame, encoding both geometry and navigation-relevant semantic information of the environment, extracted from LIDAR and image sensor data, respectively. In particular, the latter is given as an input to a fully convolutional network for semantic segmentation and the segmented image is used to label the 3D point cloud.

The work by Gonzalez and Iagnemma [59] aims at solving two key problems in terramechanics: wheel slip estimation and terrain type classification. Different architectures of CNNs are trained with grayscale images (both pre-processed or raw images) and compared against several traditional machine learning methods (i.e., SVM and MLP).

Holder and Breckon [60] propose a path prediction approach for off-road autonomous cars. Their solution can also be seen as a binary traversability prediction of the terrain. Training data are automatically created and labeled through stereoscopic visual odometry while the vehicle is driven by a human operator. The vehicle footprint is projected onto the acquired image, and those pixels actually traversed by the vehicle are marked as drivable. Eventually, the labeled data are used to train three state-of-the-art segmentation architectures.

Similarly to Maturana et al., Chiodini et al. [61] propose a solution to the semantic classification problem of voxel grid maps for Martian environments. A single channel from a stereo camera on board the rover provides an input image for a CNN. In the meantime the stereo camera information is used to sequentially build the depth map, the 3D point cloud and the voxel grid map, which is finally labeled according to the previous semantic segmentation.

Table 4 summarizes the works introduced in this section. Unless otherwise specified, by segmentation we refer to multiclass segmentation.

### 4.3. Mixed Regression and Classification

In literature some works providing a hybrid approach between terrain classification and traversal cost regression can be found. Typically, in these works a terrain classification is performed from which a traversal cost is derived as a side result, or vice versa, a map of traversal costs is built and after that a binary classification is obtained by thresholding.

#### 4.3.1. Solutions Based on Classical Machine Learning

An example of hybrid approach is given by Roncancio et al. [62] who propose a path detection method. An on-line learning of visual features (or texture descriptors) is implemented to continually update an SVM-based terrain classifier, in order to enhance the path detection as the vehicle moves. In particular, a 3D terrain traversability cost map, built from the on-board stereo camera, is combined with a terrain classification, in order to help the following segmentation of traversable region. After that, the segmentation is used again as a positive label for training the classifier.

Shan et al. [63] developed a binary traversability classification via Bayesian generalized kernel (BGK) inference [64]. The authors propose this approach to deal with the sparsity of LIDAR data acquired by a UGV. In fact a prior elevation regression through Bayesian kernel inference is performed and then, from the obtained digital elevation model, slope, roughness and step height are derived. These three features are linearly combined to obtain the traversal cost to train the model for BGK traversability inference. A continuous cost map is computed and a binary map is obtained by thresholding.

#### 4.3.2. Solutions Based on Deep Learning

Suryamurthy et al. [65] proposes a fast method for terrain semantic recognition-segmentation and roughness estimation from RGB images. The authors present a network architecture composed of a primary CNN for terrain classification and a secondary module (based on RGB features) for roughness regression. The joint pixel-wise inference of terrain labels and roughness is employed on a navigation planner of a centaur-like wheeled-legged robot in rough terrains.

An interesting training data methodology is presented by Wellhausen et al. [66]. While a quadrupedal robot is teleoperated, on-board camera images are acquired, along with foothold-terrain interaction data given by the sensorized feet. Foothold positions are projected on the collected image, taking into account the robot trajectory, and the image labels are assigned according to the features of the recorded force-torque signals from the foot. Thanks to these data, the authors are able to train a convolutional neural network capable of regressing a ground reaction score from images, and to perform semantic segmentation as well.

Zhou et al. [67] present a solution for terrain bearing and shearing prediction through vision for planetary rovers. In particular, the authors developed a two-stage process: a real-time semantic segmentation with a lightweight network, and an inference phase, predicting the terramechanical properties through Gaussian mixture model regression.

Finally, in one of our recent works [68], we explored an alternative approach in assessing environment traversability. In this case an array of traversal scores is inferred by a CNN from the images coming from a front-facing on-board camera. Assuming these images are divided into vertical bins, the array of traversal scores gives a prediction of the traversable horizon for each bin. Therefore, the traversability performed can be viewed both as a regression of continuous scores and also as a classification of the traversable terrain inside each bin. In this work a domain adaptation strategy has been adopted in order to make the network capable of generalizing from on-road to off-road unstructured environments. A proper loss-function has been designed to ensure a more conservative prediction of the traversal score.

The works presented in this section, based on both classical machine learning and deep learning, are briefly summarized in Table 5.

## 5. End-to-End Approaches

As pointed out by Grigorescu et al. concerning driverless cars, end-to-end learning systems have been mainly developed through deep learning methods. The same observation holds in the case of unstructured environments. In fact, in this context only a few solutions have been developed exploiting standard machine learning techniques. Two examples are the works by Le Cun et al. and Ostafew et al. [69,70].

Ostafew et al. [70] present a visual teach-and-repeat solution for the autonomous off-road navigation of a UGV. A feedforward action, obtained by an Iterative Learning Control (ILC) [71], is combined with a path-tracking controller while continually running along the demonstrated path.

Le Cun et al. [69] propose an early example of a convolutional neural network in order to learn a direct mapping from low-resolution images to steering commands, by training it with data collected during manual teleoperation. Therefore, although a rather “shallow" architecture is proposed, compared to current state-of-the-art CNNs, it may actually be improper to consider this solution as based on *classical* machine learning.

Moving on to recent deep learning-based solutions, Zhang et al. [72] adopt a deep reinforcement learning method based on the Asynchronous Advantage Actor-Critic (A3C) approach. The A3C architecture network takes as inputs depth images, the robot’s attitude and the elevation map of the surrounding environment. The latter is in turn built and continually updated from depth images and the full 6D robot pose, whereas robot heading is relative to a goal target location. This is necessary to locally drive the vehicle towards a goal in the unknown rough environment. A high reward is provided as the robot gets closer to or reaches the target location, whereas a negative reward is given to undesirable terminal states. The A3C architecture network processes the depth image, the elevation map and the robot orientation on three different branches, through convolutional layers and fully connected layers. These branches are then concatenated and fed into a Long Short-Term Memory (LSTM) recurrent network. The LSTM is used in order to deal better with the partially observed environment. The outputs provided as commands for a low-level controller are forward/backward motion, or turning right/left. The training of the network is performed in simulation with the dynamic model of a 4-wheel mobile platform.

In the work by Bakken et al. [73], a CNN is trained to provide a map from RGB images to three actions on the yaw, for row following in crops. The authors used a large-scale forest trail dataset and then fine-tuned their model on smaller custom datasets from agricultural settings.

An imitation learning based procedure for high-speed off-road driving tasks is proposed by Pan et al. [74], where the policy to be mimicked is provided by a model predictive controller. The learned control policy is modeled by a deep neural network made up of two sub-networks: a CNN (fed with RGB images) and a feedforward network with a fully connected layer (fed with wheel speeds). Such a control policy maps raw input sensory data to continuous-valued control actions, i.e., steering and throttle commands.

Nguyen et al. [75] propose a solution to directly map the multi-modal input sensory data to the output steering commands. A three-branched network architecture is introduced to process and fuse the three sensing modalities employed, namely 2D laser scans, and RGB-D camera data (i.e., colored images and point clouds). Laser scans are processed in order to derive a 2D occupancy map. Both the RGB image and the 2D occupancy map are given to two parallel residual net branches for visual features extraction, whereas the third branch processes the point cloud data for geometric features extraction. After that, the geometric features are concatenated with those from the RGB image branch, fed to convolutional layers and finally combined with the 2D occupancy map branch output. The steering angle is predicted from a final fully connected layer. A large-scale dataset is generated in simulation for training, thus allowing the re-creation of complex environments, such as a post-disaster scenario.

Kahn et al. [76] developed an interesting approach which can be seen as a self-supervised multi-task reinforcement learning problem combining generalized computation graphs [77] and composable action-conditioned predictors [78]. The vehicle gathers and labels, through IMU, LIDAR and wheel odometry, off-policy data experienced in the real world, including collisions. These data are used to train a predictive model of navigation-relevant events (namely collision, position, or terrain bumpiness) from the current on-board camera image and a sequence of future actions (linear and angular velocity commands). In particular, the predictive model is based on a recurrent LSTM unit. The current RGB image, passing through convolutional and fully connected layers, forms the initial hidden state of the LSTM. The recurrent unit takes as input a sequence of actions, and produces a sequence of outputs which are passed through further fully connected layers in order to predict all the events for all the future time steps. At deployment time, the trained model is combined with a user-defined reward function encoding the task required to the robot, expressed in terms of the above-mentioned events (e.g., reach a goal while avoiding collisions and/or bumpy terrain). In this manner, a planning strategy is completely defined: from current observation, the trained event-predictive model, and the reward function, a sequence of reward-maximizing actions can be planned.

A local planner based on deep reinforcement learning approach in unknown rough terrain is presented by Josef and Degani [79]. Different end-to-end architectures are discussed depending on the available range sensing system, from local height maps to no range data, namely IMU data only. The solution is tested in a dynamic simulation environment, proving the capability of planning safe local paths over surfaces with different levels of friction. A path is considered safe as long as for all positions the UGV’s roll and pitch are within two safety thresholds. The kinematic model included in this work assumes forward, right, and left actions only. When available, the elevation map is fed into convolutional layers, followed by fully connected layers. The output of this branch is concatenated with the vehicle pose and relative angle to goal (also in this case to drive the vehicle towards the goal from local data only). The concatenation serves as an input to an LSTM layer in charge of predicting the three control actions.

Manderson et al. [80] propose an architecture similar to the one in [76] for predicting terrain classes (namely smooth, rough or obstacle) over an horizon of planned future actions. In this manner, the vehicle is capable of avoiding obstacles and preferring smoother terrain areas. In particular, current images are fed to a CNN, thus forming the initial hidden state of an LSTM recurrent neural network. The model is fed with both first-person and overhead aerial images, in order to provide the network with a wider context. The two images follow two different convolutional branches. The input-output pairs of the LSTM are given by steering actions and predicted terrain class probabilities. A constant throttle is considered and, hence, not included among the control actions. At deployment time a reward function making more desirable running on smoother terrains is defined, thus allowing the planning of action trajectories that maximize the expected reward.

In Table 6 the works presented in this section are summarized (except the early works [69,70]).

## 6. Discussion

In this section the main aspects arising from a general overview of the works introduced in the previous sections will be discussed and detailed.

### 6.1. The Breakthrough of Vision: Paradigms and Sensing Technology Advancements

In his work, ref. [14] mentioned vision-based traversability approaches as those to be further explored. In fact, most of the works presented mainly relied on geometry and morphology analysis of the terrain, from the data provided by 2D or 3D laser scanners. It is apparent how in a few years the related research has dramatically moved in the opposite direction. Computer vision techniques based on deep learning have been widely assimilated and adapted to robotics, thus enabling the development of novel solutions. Therefore, classical geometric analysis has been combined with more and more robust vision-based methods for extracting high-level information.

This can also be observed in the changed sensing device arrangements of a typical unmanned ground vehicle today. Laser scanners have been placed side by side, first, to monocular cameras and, afterwards, to stereo cameras. From the technological perspective, a relevant contribution has been made by the appearance of so-called RGB-D cameras, which provide a per-pixel depth information in camera images. Based on different working principles, such as structured light or stereoscopy, most of the time these devices are now capable of providing not only RGB images with an aligned depth map, but also a 3D point cloud with color information. In some cases, the manufacturers offer software development kits that even allow real-time environment mapping, with both geometry and visual appearance. This kind of environment sensing has already been employed in several works reported in this survey [34,51,75] and is much less costly than 3D laser scanners. However, depending on the sensing technique adopted, there are some application scenarios in which RGB-D cameras cannot work whereas LIDARs can, such as in the dark. Nevertheless, we believe the current direction in environment perception is the most promising. The future trend for sensing is to move towards monocular depth estimation in order to save space at the price of a slightly increased computing power demand; this is supported by the reduction in size and cost of computing boards.

Therefore, since future exteroceptive sensors will provide more and more information regarding the environment in which a vehicle is operating, we argue that most of the research effort should be placed on the algorithms and the methodology to be adopted in processing these data. Clearly, learning-based methods will play a crucial role, thanks to their generalization capability and flexibility to deal with both types of information (namely geometry and appearance).

### 6.2. From Features to Data

As has occurred in computer vision, which is actually directly related to the advance of vision-based robotics navigation, since the introduction of deep learning attention has moved from *features* (both visual and geometric) to data. As is known, both classification and regression methods in classical machine learning use hand-crafted features extracted from sensor data which, however, are typically not easy to be defined. It is not simple even to figure out how many and which type of features are actually necessary for the classification or regression problem at hand. Hence, the initial challenge was how to define such features.

This justifies the rather limited variety of geometric features proposed for 2.5D or 3D models, based on the nearly ubiquitous principal component analysis. The most common features are terrain slope, roughness, and height step and variance. However, as underlined in [14], such features have been proposed since the work in planetary exploration by Gennery, dating back to 1999 [81]. Only in [51] a particular attention is again given to defining a different kind of features, in order to enhance terrain classification outcomes. Other interesting approaches have been presented in [72,75], where CNNs are used to extract geometric features directly from 3D point clouds, depth maps and elevation models. Newer approaches to meaningful geometric feature extraction could probably be explored, likely by exploiting deep neural networks and trying to infer those non-trivial features, through *saliency detection* [19] for instance. It is worth mentioning that there are already available in the literature solutions for complex feature extraction from point clouds through deep neural networks. Refs. [82,83,84,85] from which autonomous navigation solutions may benefit. Luckily, an increasing number of datasets with multi-modal sensor data of unstructured environments are becoming available, which proves a renewed interest in these scenarios [86,87].

### 6.3. Proprioceptive Data for Training, Exteroceptive Data for Deployment

In the past, several works have been proposed for the in-situ classification of terrain types, based exclusively on proprioceptive data while actually experiencing the traverse on the (potentially dangerous) terrain [88,89]. Among the works reported in the present survey, a recent and more frequently adopted approach consists of using proprioceptive data during training as well as for data labeling [34,35,66]. This approach has already proved to provide better classification and regression results compared to those methods based on exteroceptive data only, although only the latter are used at deployment time.

### 6.4. Learning Navigation from Experts

Learning from demonstration paradigm has found a relevant application in solving the problem of autonomous navigation in unstructured environments, both in classical machine learning and deep learning [25,28,30,32,40,41]. As already briefly mentioned, the crucial feature of these paradigms is the possibility to infer a model encoding the intrinsic motivation of the expert’s actions from sensory data, and to use such a model to perform terrain traversability regression. The obtained cost map can easily be integrated into a classical graph-search based path planning algorithm, e.g., Dijkstra, or A* [19], thus completing the sense-plan-act pipeline.

However, besides LfD-based methods, several works have performed the acquisition of training data while the vehicle was manually operated. In this manner, mainly positive examples of traversable terrain have been collected [42,43,46,60,66]. However, such an approach, which might resemble, at least conceptually, an imitation learning solution, suffers from the limited variety of acquired information. In fact, as most of these works pointed out, the training dataset is essentially biased towards positive data. Only Suger et al. [43] explicitly address this problem by proposing two specific classifier training algorithms from other application contexts with the similar issue, such as text classification [44,45]. However, we believe that framing these approaches into a true learning from demonstration paradigm may provide a better understanding of the solution resulting from the whole learning process.

### 6.5. The Importance of Simulation

As already pointed out in [18,19,90], simulation of robot behaviors, sensors and algorithms do play a crucial role in the context of robot learning. Several works among the ones in this survey extensively used simulated data to train their models [49,53,57,75]. In particular, the most beneficial aspect of simulations which has been exploited the most is the possibility to easily label training data. Clearly, this also poses the well-known *transfer domain* challenge of seamlessly deploying models trained in simulation to the real world.

This is even more important in the context of those solutions based on deep reinforcement learning, which represent an important body of the current research in this context as shown in this survey. In fact, in this case there is the need to provide (even potentially damaging) negative examples in order to obtain a robust learned policy, capable of dealing with the widest set of possible scenarios. However, the work by Kahn et al. [76] assumes the possibility of letting the vehicle experience on its own collisions while running with a random policy in the real world. Although it may sound strange, the possibility of acquiring real-world data can make the learning of the desired policy more immediate and robust. This new approach may even make the real need for simulation questionable, which has actually represented a limitation so far.

### 6.6. Multi-Robot Cooperation

Although beyond of the scope of this review, we can easily perceive how the need to combine multiple heterogeneous platforms especially with potentially different perspectives, is still strong in the context of rough environment navigation. Some works suggesting the adoption of overhead or satellite imagery have been presented [36,41,80]; others have used terrain height maps [25,29,57]. However, for some of them, it could easily be figured out that such images or elevation maps could be provided by an aerial vehicle hovering and following the ground vehicle. Several solutions have been proposed in the literature suggesting this approach for traversability [91,92,93]. As mentioned by [80], aerial data can allow a model to be trained with a higher implicit *contextual awareness*, which can definitely help the ground robot while autonomously navigating.

### 6.7. Trends and Future Challenges

#### 6.7.1. The Two Approaches: Traversability Analysis versus End-to-End Methods

Although a kind of *cognitive analysis* is absolutely needed when choosing alternative paths in unstructured environments, taking into account the difficulty and the energy expense (effort), the need to regress a *traversal cost* or to identify a *terrain type* is becoming a questionable approach, as it requires a further processing step or, more generally, a decision-making layer in the robotic navigation system.

Moreover, in the case of terrain classification, even if the significant contribution of deep learning-based segmentation is clear, it seems that few and naive solutions have been proposed to perform path planning through a segmented image. In this manner, these approaches are actually closer to solutions for computer vision problems than for real robot navigation. In [90], a wide discussion on the need for suitable metrics and evaluation for deep learning in robotics is given. However, we believe that the difficulty of identifying an effective way of actually exploiting a classified terrain type is another sign of the inadequacy of terrain traversability analysis. Furthermore, several works still propose a binary classification, which does not make any type of smart path planning possible. On the other hand, as already said, regression-based TTA methods are much more suited to path planning integration, thus making us more confident on future developments of such methods.

Conversely, recent end-to-end methods confirm there is an alternative and more integrated way to deal with navigation in unstructured environments, which seems at first glance closer to human behavior. Reinforcement learning-based methods are probably closer to this aspect, compared to other end-to-end methods, as they offer a formal paradigm for *learning from experience*. Unsurprisingly, these methods have been proposed in several works [72,76,79,80]. The main limitation of these approaches is related to the need of performing on board real-time computing in order to reactively control the vehicle. Some TTA-based methods operate *offline* on the environment model instead, thus relaxing the timing constraints.

Still thinking of human behavior, it could be interesting to investigate how the cognitive processes related to navigation actually occur in humans, to try to gain an insight of new paradigms to be explored. Some preliminary results have been achieved in this sense [94,95,96]. However, it may be also limiting to think only of ground vehicles with the same navigation capabilities as humans. In fact, there already exist cameras capable of perceiving better than human eyes [97], event-based cameras [98,99], a completely different paradigm of vision system to classical intensity-based cameras. There are also radars capable of penetrating the ground [100] and some early solutions for traversability analysis have been proposed [101]. Therefore, it is not completely unrealistic to imagine field robots capable of locating traversable paths outperforming human capabilities.

#### 6.7.2. Platform Independence and Generalization

An important issue is related to the level of independence of the proposed solutions from the specific platform, or, in other words, how generalizable such solutions are. In this sense, end-to-end methods suffer from limited generalization, e.g., when specific steering systems must be considered [60]. It seems we are still some way from a solution general enough to be deployed regardless of the type of vehicle. However, it is worth remarking that the concept of traversability, according to the definition in [14], seems to be inherently related to the specific type of vehicle. This aspect underlines how unsuitable such a concept is for generalization.

Faigl and Prágr [34] emphasised the need to define generalizable traversal costs and, with this idea, they considered instantaneous power consumption, maximum achievable forward velocity, and attitude stability. These measures can be used regardless of the specific type of platform. An impressive proof of the potential *generalizable* solutions obtainable from learning, in the context of locomotion, is presented in [102]. Without using any exteroceptive data, the agent is trained through reinforcement learning to control joints in position from IMU data and joints encoders, thus being able to acquire core locomotion skills for legged and wheeled/legged platforms. We wish higher level navigation tasks could also be solved with highly generalizable solutions.

## 7. Conclusions

With the present review, we provided a broad high-level overview of the general problem of autonomous navigation of ground vehicles in unstructured environments. Different types of approaches have been presented, at different levels of the navigation pipeline (namely environment sensing, planning, control), all of them sharing the unstructured nature of the considered environment. Although these approaches might look rather distant in some cases, we hope this paper could promote a kind of combination of the separate efforts.

We focused mainly on learning-based methods as we found that most of the attention of current robotics research is focused on indoor service robots, self-driving cars in urban scenarios or, in general, on structured environments. We believe there is still a gap between the deployment of robots in structured and in unstructured environments, and we argue that the latter are those scenarios actually useful for the most relevant real world applications of field robotics.

## Figures and Tables

**Table 1:** Works on regression of traversal costs with classical machine learning.

	Learning Method	Input Data	Vehicle Locomotion	Traversal Cost
Ratliff et al., 2006 [28] (1)	boosted MMP	2D laser scans, RGB and NIR images	not specified	learned non-linear function
Ratliff et al., 2006 [28] (2)	boosted MMP	height maps	legged	learned non-linear function
Kolter et al., 2008 [29]	hierarchical IRL	height maps	legged	learned linear function
Silver et al., 2010 [32]	non-linear MMP	2D laser scans, RGB images	wheeled	learned non-linear function
Zucker et al., 2011 [25]	ranking SVM	height maps	quadruped	learned exponential function
Faigl and Prágr, 2019 [34]	self-organizing maps	RGB-D camera image and depth	legged	power consumption, max forward velocity, attitude stability
Bekhti and Kobayashi, 2020 [35]	GPR	RGB images	wheeled	vibration
Quann et al., 2020 [36]	GPR	satellite imagery	wheeled	power consumption

Maximum Margin Planning (MMP); Inverse Reinforcement Learning (IRL); Support Vector Machine (SVM); Gaussian Process Regression (GPR); Near Infrared (NIR); Red Green Blue (RGB).

**Table 2:** Works on regression of traversal costs with deep learning.

	Learning Method	Network Architecture	Input Data	Vehicle Locomotion	Traversal Cost
Zhang et al., 2018 [39]	ME-DIRL	FCN	3D laser scans, RGB images	wheeled	undefinable
Zhu et al., 2019 [40]	ME-DIRL	FCN	3D laser scans	wheeled	learned reward function
Pflueger et al., 2019 [41]	IRL with soft VIN	FCN	satellite imagery	wheeled	learned reward function

Maximum Entropy Deep Inverse Reinforcement Learning (ME-DIRL); Value Iteration Network (VIN); Fully Connected Network (FCN).

**Table 3:** Works on classification of terrain type with classical machine learning.

	Classification Method	Input Data	Vehicle Locomotion	Type of Classification
Santamaria-Navarro et al., 2015 [42]	GPC	3D laser scans	wheeled	binary
Suger et al., 2015 [43]	POS [44], PNB [45]	3D laser scans	wheeled	binary
Sock et al., 2016 [48]	SVM	3D laser scans, RGB images	wheeled	binary
Lee et al., 2017 [46]	kNN	RGB images	not specified	binary
Ahtiainen et al., 2017 [47]	SVM	3D laser scans	wheeled	binary
Hewitt et al., 2017 [49]	MLP	2D laser scans	wheeled	binary
Deng et al., 2017 [50]	GPC	RGB images	wheeled	binary
Schilling et al., 2017 [54]	RF	3D laser scans, RGB images	wheeled	multiclass
Bellone et al., 2018 [51]	SVM	RGB-D camera point cloud	wheeled	binary
Kingry et al., 2018 [52]	MLP	RGB images	undefined	multiclass
Martínez et al., 2020 [53]	several	3D laser scans	wheeled	binary

Gaussian Process Classification (GPC); Support Vector Machine (SVM); k-nearest neighborhood (KNN); Multilayer Perceptron (MLP); Random Forest (RF).

**Table 4:** Works on classification of terrain type with deep learning.

	Classification Method	Type of Network	Input Data	Vehicle Locomotion
Rothrock et al., 2016 [55]	semantic segmentation	FCN	RGB images	wheeled
Valada et al., 2016 [56]	semantic segmentation	FCN	RGB stereo and NIR images	wheeled
Chavez-Garcia et al., 2018 [57]	binary classification	CNN	height maps	wheeled
Maturana et al., 2018 [58]	3D semantic classification	FCN	3D laser scans, RGB images	wheeled
Gonzalez and Iagnemma, 2018 [59]	multiclass image classification	CNN	grayscale images	wheeled/tracked
Holder and Breckon, 2018 [60]	binary segmentation	FCN, CNN	RGB images	wheeled
Chiodini et al., 2020 [61]	3D semantic classification	CNN	RGB stereo images	wheeled

Convolutional Neural Network (CNN).

**Table 5:** Mixed classification/regression methods for terrain traversability.

	Method	Input Data	Vehicle Locomotion
Roncancio et al., 2014 [62]	on-line retrained SVM	RGB stereo images	wheeled
Shan et al., 2018 [63]	BGK inference	3D laser scans	wheeled
Suryamurthy et al., 2019 [65]	semantic segmentation and pixel-wise regression	RGB images	wheeled-legged
Wellhausen et al., 2019 [66]	regression and segmentation via CNN	RGB images	legged
Zhou et al., 2020 [67]	semantic segmentation, GMM regression	RGB images	wheeled
Palazzo et al., 2020 [68]	regression and segmentation via CNN	RGB images	tracked

Generalized method of moments (GMM).

**Table 6:** Works on end-to-end deep learning based navigation approaches.

	Learning Method	Type of Network	Input Data	Output Command	Vehicle Locomotion
Zhang et al., 2018 [72]	DRL	FCNs, LSTM	depth images, 3D robot pose	steering angle	wheeled
Bakken et al., 2019 [73]	supervised learning	CNN	RGB images	yaw command	not defined
Pan et al., 2020 [74]	imitation learning	CNN, FCN	RGB images	steering angle and throttle	wheeled
Nguyen et al., 2020 [75]	supervised learning	three-branch deep neural network	RGB-D camera images, point clouds, 2D laser scans	steering angle	not defined
Kahn et al., 2020 [76]	self-supervised multi-task DRL	CNN, LSTM	RGB images	angular and linear velocity	wheeled
Josef and Degani, 2020 [79]	DRL	CNN, LSTM	IMU data, height maps	forward, right, left motion	wheeled
Manderson et al., 2020 [80]	self-supervised DRL	CNN, LSTM	RGB images	steering angle	wheeled

Long Short-Term Memory (LSTM).

## Abbreviations

The following abbreviations are used in this manuscript:

CNN	Convolutional Neural Network
FCN	Fully Connected Network
GPC	Gaussian Process Classification
GPR	Gaussian Process Regression
DIRL	Deep Inverse Reinforcement Learning
DRL	Deep Reinforcement Learning
IMU	Inertial Measurement Unit
IRL	Inverse Reinforcement Learning
kNN	k-nearest neighborhood
LfD	Learning from Demonstration
LIDAR	Light Detection And Ranging (s)
LSTM	Long Short-Term Memory
ME-DIRL	Maximum Entropy Deep Inverse Reinforcement Learning
MLP	Multilayer Perceptron
MMP	Maximum Margin Planning
NIR	Near Infrared
PCA	Principal Component Analysis
RF	Random Forest
RGB-D	Red Green Blue and Depth (camera)
SVM	Support Vector Machine
TTA	Terrain Traversability Analysis
UGV	Unmanned Ground Vehicle
VIN	Value Iteration Network
